# Enhancements and Challenges in CoAP—A Survey

**DOI:** 10.3390/s20216391

**Published:** 2020-11-09

**Authors:** Muhammad Ashar Tariq, Murad Khan, Muhammad Toaha Raza Khan, Dongkyun Kim

**Affiliations:** 1 Department of Artificial Intelligence, Kyungpook National University, Bukgu, Daegu 41566, Korea; tariqashar@knu.ac.kr; 2 School of Computer Science and Engineering, Kyungpook National University, Bukgu, Daegu 41566, Korea; mkhan@knu.ac.kr (M.K.); toaha@knu.ac.kr (M.T.R.K.)

**Keywords:** CoAP, IoT, WSN, congestion control, enhancements in CoAP

## Abstract

The Internet of Engineering Task (IETF) developed a lighter application protocol (Constrained Application Protocol (CoAP)) for the constrained IoT devices operating in lossy environments. Based on UDP, CoAP is a lightweight and efficient protocol compared to other IoT protocols such as HTTP, MQTT, etc. CoAP also provides reliable communication among nodes in wireless sensor networks in addition to features such as resource observation, resource discovery, congestion control, etc. These capabilities of CoAP have enabled the implementation of CoAP in various domains ranging from home automation to health management systems. The use of CoAP has highlighted its shortcomings over the time. To overcome shortcomings of CoAP, numerous enhancements have been made in basic CoAP architecture. This survey highlights the shortcomings of basic CoAP architecture and enhancements made in it throughout the time. Furthermore, existing challenges and issue in the current CoAP architecture are also discussed. Finally, some applications with CoAP implementation are mentioned in order to realize the viability of CoAP in real world use cases.

## 1. Introduction

Wireless Sensor Networks are used widely in many applications such as in the Internet of Things (IoT) domain, Internet of Underwater Things (IoUT), Internet of Everything (IoE), etc. The nodes in wireless sensor networks (WSN) are interconnected to each other for the purpose of monitoring, detecting and gathering data from environments and communicating it among different nodes or propagating it to a data collection point [1]. These nodes are usually equipped with limited memory, low battery power, and constrained processing capabilities. Moreover, these devices are typically employed in low bit error rate environments with lossy communication link. The limitations of these devices and communication links requires a lighter and reliable application protocol with an efficient congestion control mechanism for IoT and WSNs.

**Figure 1:**
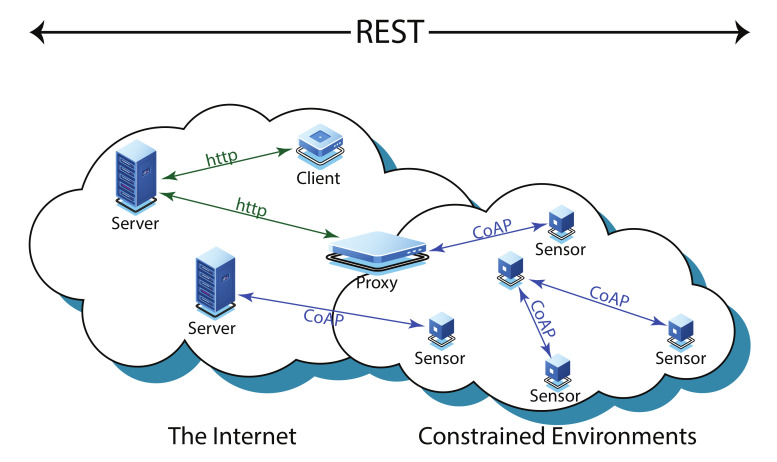
An overview of CoAP architecture.

To fulfil the need for a lighter application protocol for IoT devices a specialized web transfer protocol called the Constrained Application Protocol (CoAP) was developed by IETF for low power constrained network devices [2]. The CoAP functionality is based on the REST (Representational State Transfer) architecture [3]. Figure 1 represents an overview of the CoAP architecture. The transport layer protocol in CoAP is User Datagram Protocol (UDP). Unlike the Transmission Control Protocol (TCP), UDP is unreliable and vulnerable to congestion in network [4]. Therefore, a congestion control mechanism is required in CoAP. A default congestion control mechanism is established for CoAP [2], which uses a simple binary exponential backoff (BEB) [5]. The default mechanism of CoAP, however, is not efficient or effective. To overcome the issues in default congestion control mechanism of CoAP, a number of other schemes have been developed such as CoCoA [6], CoCoA+ [7], pCoCoA [8], etc. These schemes are further discussed in detail in the upcoming sections. Since default congestion control mechanism hardly fulfils the requirements of WSNs and IoT networks, another congestion control mechanism named as Congestion Control/Advance (CoCoA) is standardized by IETF [6]. The CoCoA provides better congestion control mechanism for CoAP with minimal additional resources; however, several problems in CoCoA are also detected which causes it to work even worse than default congestion control mechanism of CoAP under various network conditions [7]. This led to the development of number of advance congestion control mechanisms for CoAP to overcome the shortcomings of default CoAP and CoCoA. Most of these methods are for reliable communication in CoAP, whereas some are presented for unreliable communication as well. These solutions are based on Round Trip Time (RTT) calculations, queueing delay, traffic rate conditions, and bandwidth delay product.

Along with enhancements in congestion control mechanism of CoAP, research was done to enhance CoAP in other domains as well. CoAP being a new protocol is not fully explored in many domains. As it is being employed in different applications, more and more application specific enhancements are being performed. Much research was carried out to enhance CoAP for security, end-to-end authentication, streaming services, etc. The details of these mentioned enhancements in CoAP are presented later in this paper. In order to highlight the use of enhanced versions of CoAP congestion control schemes, the survey also provides the qualitative and quantitative analysis of these schemes. The analysis provides clear insight of average percentage improvements in various performance metrics of proposed schemes compared to the default congestion control mechanism of CoAP.

The presented survey discusses the enhancements in CoAP in an application-oriented manner. Due to the increased demands and diverse requirements of the IoT communication solutions, the survey first introduces an overview of CoAP protocol in Section 2 following with an overview of applications of CoAP in Section 3. The applications of CoAP are also briefly summarized in Table 1. Section 4 highlights evolution and enhancements in CoAP focusing mainly on congestion control mechanisms of CoAP. Table 2 represents the comparison of various techniques of congestion control in default and advance CoAP versions. The qualitative and quantitative analysis of the enhanced congestion control schemes is presented in the Section 5. Table 3 summarizes the quantitative analysis of enhanced congestion control schemes. Section 6 illustrates some open challenges and research directions. Finally, the concluding remarks are presented in Section 7.

## 2. Constrained Application Protocol (CoAP)

**Figure 2:**
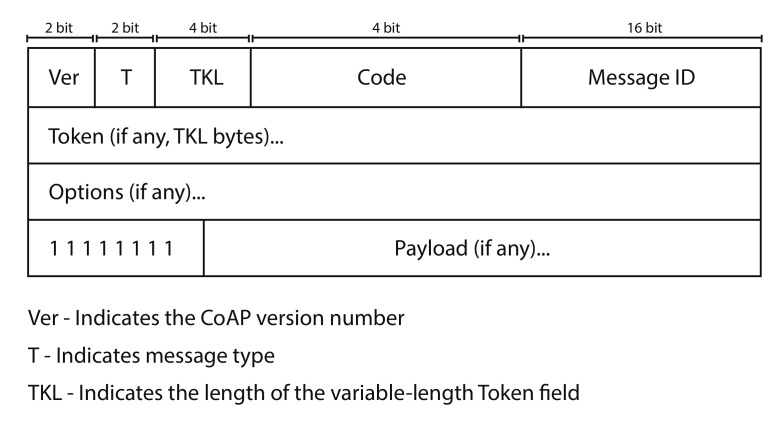
CoAP Message Header.

For the purpose of achieving lightweight packet exchanges between constrained IoT devices, IETF developed a lightweight application layer protocol called CoAP. Similar to HTTP, CoAP is based on REST architecture and uses GET, POST, PUT, and DELETE request methods. The CoAP has a small and fixed header length of 4 bytes which includes optional parameters of token, options, and payload. The CoAP header is shown in Figure 2. CoAP provides request/response model as well as publish/subscribe model for resource observation. A CoAP client sends a request to server in a request/response model using RESTful methods and server responds to it. For resource observation [33] the client subscribes to server resource for some time period and the server updates the client only when there is some change or update in the resource. This is helpful in saving energy in many IoT scenarios as client does not have to request state of server resource constantly.

There are four types of messages that are supported by CoAP:

1. Confirmable (CON)
2. Non-confirmable (NON)
3. Acknowledgement (ACK)
4. Reset

**Figure 3:**
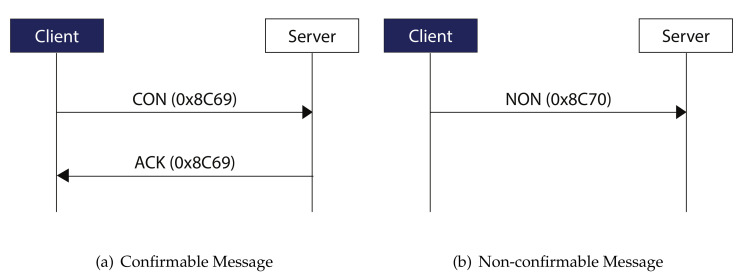
Examples of Confirmable and Non-confirmable CoAP messages.

Moreover, CoAP supports two modes of transmission: reliable and non-reliable. For reliable communication, CON message is sent from one node to another and the sender requires the ACK response. Whereas unreliable communication uses NON messages which do not required any ACK from receiver. In case of reliable transmission, if the sender does not receive ACK response from receiver in a particular period of time known as retransmission timeout (RTO), the sender retransmits the packet. These retransmissions cannot increase the MAX_RETRANSMIT which is usually set to 4. Examples of CON and NON messages are represented in Figure 3.

**Figure 4:**
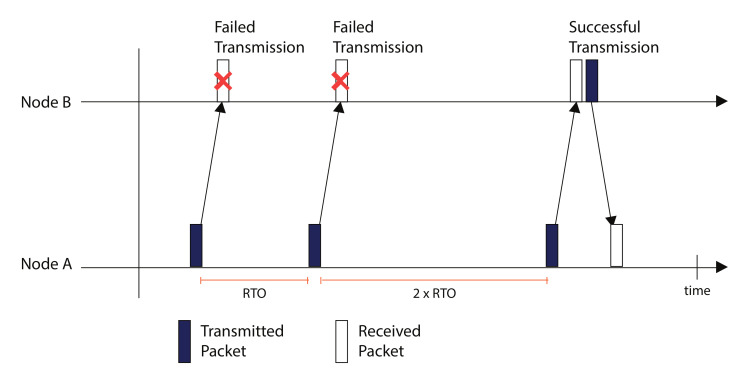
CoAP Default Congestion Control.

For congestion control, CoAP has a default congestion control mechanism which is based on BEB. Initially for first transmission the RTO value is set to 2 to 3 secs and this value is doubled on expiration for each retransmission until the sender is out of total number of retransmissions. However, the default congestion control mechanism of CoAP is not very efficient and does not correspond to dynamic network conditions. Therefore, many enhancements in congestion control are developed over time which are discussed in the later sections. The default congestion control mechanism of CoAP is shown in Figure 4.

Unlike HTTP, CoAP runs over UDP. This helps in avoiding costly handshakes as in case of TCP. CoAP also allows UDP broadcast and multicast for addressing [34]. Owing to the use of UDP in CoAP, large sized payloads can be transferred using block-wise transfer [35] where the resource is split into smaller pieces and sent over multiple CoAP messages. For transferring data securely in CoAP, it uses Datagram Transport Layer Security (DTLS) on top of UDP protocol [34].

Owing to its lightweight capability along with secure reliable and non-reliable communication modes and resource observation model, CoAP has gained immense popularity in IoT and WSN applications. Its applications range from home automation to healthcare management systems. Some applications of CoAP are mentioned in detail in Section 3.

## 3. Applications of CoAP

The CoAP has found its application in the various fields. It is being used to provide the interoperability and integration services, authentication and authorization services, streaming services and so on. The implementation of CoAP in various domains has enabled us to achieve secure, fast, and efficient communication between devices. This section briefly summarizes some applications of CoAP and the services it provides.

### 3.1. Interoperability and Integration

This subsection describes how the CoAP is used for the purpose of interoperability and integration. The CoAP is implemented for interoperability with other application layer protocols such as HTTP and MQTT as well as integration of different healthcare standards.

#### 3.1.1. Interoperability with HTTP

Ref. [9] works on the compatibility of mobile healthcare platforms with internet and world wide web. It devises a smartphone proxy prototype based on CoAP which enables direct compatibility of medical sensors with internet along with interaction with other nodes using RESTful communication. The patient’s medical data can be directly communicated from patient’s smartphone proxy to doctor’s smartphone if it supports CoAP. The medical sensor data can also be transferred to the medical centers or doctors via internet where the smart phone acts as proxy between client(patient’s smartphone) and server(doctor’s smartphone), implementing HTTP and CoAP conversion in order to increase the compatibility. Moreover, the CoAP observe feature reduces the overhead of constant communication of server with client for gathering medical sensor data. Instead, it only receives periodic responses from the smartphone proxy. CoAP being a lighter IoT protocol also helps in implementing this architecture without much use of CPU, power, and memory resources.

#### 3.1.2. CoAP Interoperability with MQTT for Healthcare

In [10], Oryema et al. design and implement a messaging system for IoT devices used in healthcare sector. Although the use of Message Queuing Telemetry Transport (MQTT) protocol in IoT devices has advantages of supporting many-to-many communication and with a small overhead; however, the limited rest time of devices due to constant wait for request is disadvantageous for these constrained devices. Therefore, the proposed system implements the messaging system with MQTT model using CoAP. The architecture consists of publisher, subscriber, and message broker where publisher and subscriber are CoAP clients and broker is a CoAP server. The use of CoAP observer method is employed to implement the subscription method of MQTT and GET, POST, PUT and DELETE methods are used for topic resource discovery, topic resource registration, publishing measured medical data, and resource deletion respectively. The use of CoAP in implementing the MQTT based model is beneficial for keeping the advantages of MQTT protocol as well as providing rest to constrained IoT devices enabling them to consume minimum energy and drain less battery.

#### 3.1.3. Interoperability with Healthcare Platform

CoAP is employed in [11] to design and implement an international healthcare platform in conjunction with IEEEE 11073 PHD [36] which is a healthcare standard developed for interoperability between medical devices and systems. An IoT device in the proposed architecture has a CoAP server, healthcare data collector and a state manager. The CoAP server performs the roles of (i) courier i.e., it handles all the requests and responses to the server, (ii) manager i.e., it performs the task of maintaining resource list, and (iii) coordinator i.e., provides harmony between state manager and data collector. The healthcare data collector is responsible for collecting data through the physical layer and converting it to IEEEE/ISO 11073 PHD standard format. Finally, in the physical layer, the state manager controls the IoT device’s states. The proposed system enables the application of ISO/IEEE 11073 DIM/Service Model integrated with CoAP for IoT environments.

#### 3.1.4. Integration of Healthcare Standards

A design and implementation of a communication system is presented in [12] by using CoAP. The CoAP is employed in the integration of two healthcare standards ISO/IEEE 11073 and IHE PCD-01 for communicating between medical IoT devices such as medical sensors attached to patients and hospitals. CoAP RESTful methods are used to realize the CoAP communication. Moreover, authors have also compared the performance of CoAP in this application with MQTT and HTTP. The results exhibit faster transactions from CoAP with fewer internet resources used comparatively.

#### 3.1.5. Integration with OSGP

Open Smart Grid Protocol (OSGP) is the communication protocol used for information exchange between devices in Smart Grids (SG). However, OSGP does not support integration with constrained IoT devices running CoAP which is used for communication in most IoT devices. In order to map the data packets between CoAP and OSGP, [13] proposes a solution called CoAP and OSGP Integration for the Internet of Things (COIIoT). A function mapping layer is applied to each request/response individually. CoAP uses MicroCoAP library to execute a GET method and this message is then mapped to an OSGP partial read request. The request type, message id and packet size are extracted from the CoAP packets and analyzed for match between CoAP request and OSGP request. Afterwards, the two fields of OSGP packet (count and offset) are filled with the CoAP message payload. The count field is embedded with message size whereas the offset field includes message contents. For the reverse mapping, the Pending Event Descriptor (PED) holds the OSGP packet and is included in options field of CoAP packet and the payload size field of CoAP is filled with count value of OSGP packet and the payload field of CoAP is filled with offset/data value of OSGP packet. However, performing mapping for each request/response individually may add additional overhead and latency, therefore, it needs to be experimentally evaluated.

### 3.2. Security

Security is a critical issue in any application. The CoAP is used in the implementation of security services such as authentication, authorization and access control. The details of these implementations are mentioned in this subsection.

#### 3.2.1. Authentication and Authorization

The authors in [14] have used the CoAP for the purpose of integration between use of Authentication, Authorization and Accounting (AAA) infrastructures and Extensible Authentication Protocol (EAP). Being a lightweight transport protocol, the use of CoAP covers the restrictions of Low-Power Wide Area Networks (LP-WAN) such as constrained bandwidth, low memory, and restricted resources in terms of CPU and energy consumption. The proposed scheme named as Low-Overhead CoAP-EAP (LO-CoAP-EAP) provides a solution for network access authentication for constrained devices in LP-WAN. CoAP in this scheme enables the design of a network authentication service which can be used independently and does not depend on any LP-WAN technology. The CoAP is used for communication between the constrained devices. The architecture contains (i) a smart object acting as a CoAP server, (ii) a controller as CoAP client and (iii) a AAA server. The exchange of CoAP POST messages is employed to obtain the identity of the smart object for providing it with the required services.

#### 3.2.2. Authentication and Access Control

Tamboli et al. propose a CoAP-based authentication and access control framework for IoT in [15]. A low power security framework is proposed for main server and per service-based fine grain access control is implemented. CoAP is implemented to provide communication with packets with low overhead for the IoT environment. The scheme employs authentication and access control schemes such as Kerberos with CoAP and optimized ECDSA is issued for encryption and privacy. Ticket generation-based solution is provided for authentication and accessing services. The client is issued a valid ticket for authentication upon registration, which is then used for gaining access control while requesting a particular service from main server.

### 3.3. Streaming Services

The blockwise transfer capability of CoAP enables the use of CoAP in the streaming services such as media streaming or video streaming. The application of CoAP in streaming services is mentioned further.

#### 3.3.1. Media Streaming

For media streaming services, [16] proposes a dynamic streaming over CoAP (DASCo). The algorithm uses the formats of dynamic streaming over HTTP (DASH) and employs CoAP for delivering the media segments to consumers. Some modifications are also made in default CoAP for DASCo to make the streaming service more efficient. The consumer’s application requests the media segments consecutively by using the GET method of CoAP. CoAP’s stop-and-wait data transfer can also help in downloading several media segments in parallel by changing the NSTART parameter of CoAP. The block-wise transfer is also useful in determining the progress of download and knowing when to stop it. Finally, to achieve delay sensitive live streaming, MAX_RETRANSMIT parameter of CoAP can be enhanced to get more retransmissions.

#### 3.3.2. Video Streaming Services

Another video streaming technique is performed over CoAP in [17]. The proposed scheme adaptively changes the quality of video based on the available throughput, download time of the segment and size of the video segment for next video segment. In order to achieve high quality video streaming without experiencing any interruptions and needless quality switches, the CoAP client in the model selects the video rate from a set of video rates available on the CoAP server, for each segment of video. This rate is selected by estimating the throughput of next segment which is calculated using throughput variations in previously downloaded segments. The main reason to employ CoAP is the use of CoAP in video streaming services for constrained wireless environments.

### 3.4. Cloud Computing Services

Integrating cloud with IoT broadens the aspect of IoT applications. The lightweight IoT protocol, CoAP, enables the integration of IoT nodes with cloud where CoAP performs data transmission between IoT sensor nodes and cloud.

#### CoAP Integration with Cloud

The IoT data transfer implementation between raspberry pi and the cloud is achieved by Scott et al. in [18], based on CoAP. The proposed system consists of (i) sensors, (ii) raspberry pi, (iii) CoAP-based gateway and (iv) cloud platform. The CoAP is implemented in transferring the data from sensors, which act as IoT nodes, to cloud platform. Humidity and temperature sensors are used in the system and their data formatted in JavaScript Object Notation (JSON) is periodically transmitted to ThingsBoard cloud endpoint using CoAP POST messages.

### 3.5. Resource Observation and Discovery

This subsection mentions the role of CoAP in implementing the resource observation and resource discovery operations in [19,20] respectively.

#### 3.5.1. Resource Observation

**Figure 5:**
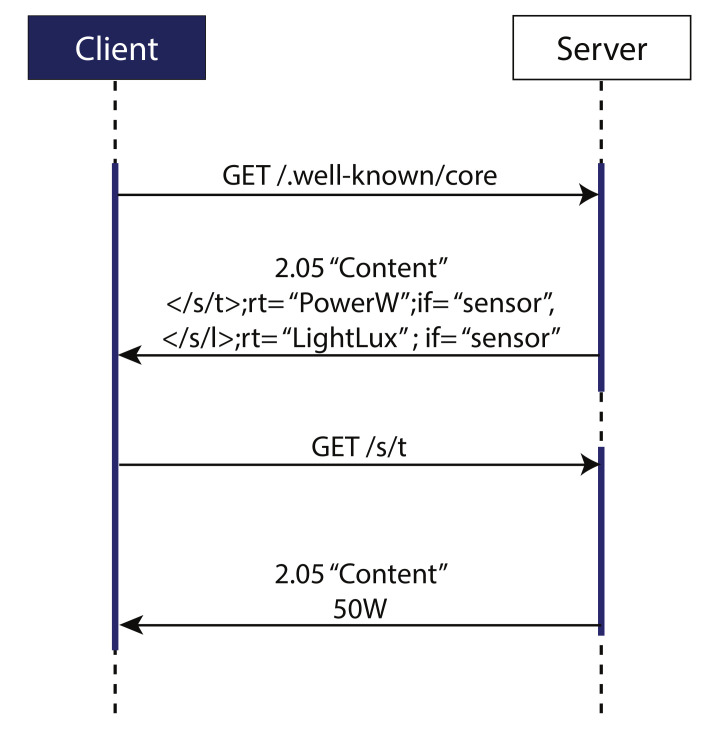
CoAP Resource Observation

Ref. [19] describes briefly about using CoAP protocol for observing resources in IoT environment and WSNs. A simple application of observing the temperature form a temperature sensor is exhibited. The temperature sensor acts as a CoAP server which can be queried by any CoAP client to observe the resource i.e., temperature in this case. The CoAP client can exploit the use of RESTful methods for query. An example of how resource observation is implemented in CoAP is shown in Figure 5. Here a CoAP client sends a request a CoAP server as an observer of a particular resource i.e., value of power in wattage in this case. This establishes an observational relationship between CoAP client and the resource. The client is then provided with the current state of the resource in response to the GET request generated by client.

#### 3.5.2. Resource Discovery

A hybrid resource discovery mechanism is proposed in [20] using CoAP. The paper introduces discovery of resources using a hybrid approach which can switch between centralized/distributed resource discovery mechanisms. It is based on the previously developed Proactive Resource Directory (RD) Discovery mechanism proposed in [37]. The centralized approach is called the Proactive RD Discovery (PRD) and the distributed is called push-pull resource discovery. In PDR, presence of RD is advertised in network using CoAP POST messages and nodes can cache this information for a specified lifetime. This information can also be updated with PUT or POST CoAP messages. When a CoAP client needs to access a RD, it can generate a CoAP GET request and get it from nearby cached node. Fully distributed push-pull Resource Discovery (FDR) is employed when the RD is absent in nodes of network. In FDR, some nodes in the network proactively advertise (pushes) their cached resources based on the algorithm proposed in [38]. Moreover, FDR forwards the on-demand requests by pull mode. The CoAP GET method is employed to demand the RD in pull mode of FRD.

### 3.6. Real-Time Remote Monitoring

Another application of CoAP is the real time remote monitoring of data in the domain of healthcare. The subsection describes how CoAP is used for real time remote monitoring.

#### Remote Monitoring and Real-Time Display in Healthcare

Another application of CoAP in implementing a remote healthcare monitoring system is presented in [21]. The proposed approach can monitor the vitals of patient and display them in real time using a web browser. The CoAP protocol is implemented in the Mozilla Firefox Web browser which acts as a client and the sensors attached to patient’s body act as CoAP server. Two implementations of CoAP are performed in simulating the proposed approach: Erbium which is a REST service for Contiki OS and Copper which is an add-on for the Mozilla browser. The web browser acting as a CoAP client with Copper add-on implementation uses the GET method of CoAP to access the resources from server.

CoAP is implemented in various domains for applications such as interoperability and integration with other protocols and standards, streaming services, security, resource observation and discovery and many other. Section 3 describes the use of CoAP in these applications; however, the basic architecture of CoAP has many limitations that need to be fulfilled. For example, the congestion control mechanism in basic CoAP is inefficient and does not perform well in dynamic network conditions, which led to the development of several congestion control mechanisms proposed by researchers. Similarly, the limitations of interoperability of CoAP with other protocols for instance the interoperability of CoAP with HTTP for cross protocol authentication was not possible in basic CoAP. Moreover, the basic CoAP runs on UDP and uses DTLS for security. The DTLS is not a very secure protocol and is vulnerable to hacking attacks. In addition, high cost and computational power requirements of DTLS also make it less suitable. Hence, to make CoAP more secure, various enhancements in the CoAP security are proposed which include enhancements in the DTLS protocol, use of hashing techniques for CoAP security instead of using DTLS, etc. For using the CoAP for streaming services in constrained devices, basic CoAP employs the stop-and-wait mechanism to send packets which is an inefficient method for sending content between nodes. Therefore, here again there is a need for enhanced version of CoAP for employing CoAP in streaming services. To fulfil the limitations in basic CoAP for various applications, numerous enhancements in the architecture of basic CoAP are proposed by the researchers. The enhancements are described in Section 4 in detail.

## 4. Enhancements in CoAP

CoAP is being used in WSNs and IoT environments in various domains. The implementation of CoAP in different applications has highlighted some shortcomings in the basic architecture of CoAP. Therefore, various enhancements have been made in the CoAP architecture. The current section discusses in detail the enhancements made in CoAP congestion control mechanism, CoAP security, CoAP interoperability and so on.

### 4.1. Congestion Control Mechanisms in CoAP

This section covers congestion control mechanisms presented in default CoAP and a numerous other advance congestion control mechanism for CoAP based on RTT measurements, gradients of RTT measurements, bottleneck bandwidth of network link, traffic rates or packet loss ratio.

#### 4.1.1. Default CoAP

Congestion Control in Default CoAP: The default CoAP employs a BEB method to cater the congestion control in the network [2]. In case of reliable transmission, a CON message is transmitted from a client node to a server node. If the message is not successfully transmitted in the first attempt, a retransmission is carried out. The CoAP choses a random value of RTO for the first transmission between 2–3 s. If the first retransmission is failed, the BEB doubles the RTO in order to avoid congestion. Hence the new RTO $\left(RTO_{new}\right)$ value is twice the previous RTO $\left(RTO_{prev}\right)$ value according to Equation (1).

(1) $$\begin{array}{c} RTO_{new}=2*RTO_{prev} \end{array}$$

This method of congestion control is not very efficient as it causes long idle delays in the network and does not consider the dynamic network conditions. To make it efficient, an advance congestion control mechanism named as Congestion Control/Advance (CoCoA) was developed.

#### 4.1.2. CoCo-RED

The default CoAP does not effectively performs group communication and observe resources. Therefore, a congestion control mechanism for observe group communication is presented in [22], named as Congestion Control Random Early Detection (CoCo-RED). The main components of the scheme include:

1. Determination and calculation of RTO timer
2. Management using Revised Random Early Detection (RevRED) algorithm for avoiding congestion
3. Fibonacci Pre-Increment Backoff (FPB) algorithm for implementing backoff timer

The CoCo-RED initially sets the RTO value randomly between 2 to 4 s and uses FPB in case of retransmissions to set the RTO. To avoid congestion by Buffer Management Technique (BMT), the proposed mechanism works dynamically and uses RevRED to calculate the network density based on the average queue size (AvgQ). The RevRED algorithm drops the arriving packet before client’s buffer queue overflows. The AvgQ size is calculated using the exponential weighted moving average. The algorithm works according to the following principles:

1. If AvgQ < Min threshold, arriving packet is placed in queue
2. If Min threshold < AvgQ < Max threshold, arriving packet is dropped based on the dropping probability formula presented by the proposed method
3. If AvgQ > Max threshold, arriving packet is dropped based on the exponentially dropping probability formula presented by the proposed method

In case packets are dropped due to congestion, retransmissions are carried out. For calculating the RTO for packet retransmissions, FPB is employed. For each retransmission, the FPB is multiplied with previous value of RTO for finding new RTO value for next retransmission. The FBP uses Fibonacci numbers to multiply with previous RTO in each retransmission, which achieves lower RTO value compared to Binary Exponential Backoff of default CoAP in each subsequent retransmission.

CoCo-RED helps to reduce the response time of network in addition to packet loss. However, the backoff values are fixed and do not vary according to the dynamic conditions of network.

#### 4.1.3. CoCoA

To overcome the problems in default CoAP congestion control, CoCoA introduces an adaptive RTO calculation feature in addition to a Variable Backoff Factor (VBF) instead of BEB and an RTO aging mechanism. CoCoA defines two RTO estimators: a weak RTO and a strong RTO estimator [6]. The strong RTO estimator calculates the RTO values for the next message transmission based on the measurement of strong RTT whereas the weak RTO estimator calculates the RTO values based on the weak RTT. The strong RTT is the RTT value obtained after the first successful transmission while the weak RTT value is the RTT value obtained after at least one retransmission. The calculation for the final overall RTO is based on the values of weak and strong RTT and RTO values. For detailed calculations of overall RTO, the reader is referred to [6].

For the purpose of backoff value CoCoA applies a VBF according to the network status information in order to avoid long idle delays. Depending on the initial RTO value of a transmission, a VBF is applied to retransmissions. If initial RTO is very small i.e., less than 1 sec, a larger VBF is applied and for large RTO value i.e., greater than 3 s, a smaller backoff is applied. For a value in between 1 and 3 s, the value of VBF is set to an optimum value of 2, in correspond to BEB. The values of VBF are mentioned in [6].

Another issue concerning the aging of RTO values is covered by CoCoA. The RTO aging mechanism is applied in the case of small and large RTO estimations. For an RTO estimation value below 1 s and above 3 s, if new RTT measurements are not made for 16 or 4 times the current RTO value respectively, CoCoA uses the RTO aging mechanism and changes the RTO value in a way that it approaches the default initial value.

The CoCoA outperforms default CoAP congestion control mechanism; however, there is an ambiguity in the weak RTT estimator calculation. Moreover, the weak RTT value may vary in each consecutive calculation as the it is unknown that after how many retransmissions does the ACK of a message is received. This leads to long overall RTOs and in turn, high idle delays.

#### 4.1.4. Four-State Estimator Scheme

Since CoCoA is unable to distinguish which retransmission the received ACK belongs to, it performs the RTO calculations based on the original start time which causes idle delays between successive transmissions. To overcome this issue, the authors in [23], have proposed a 4-state estimator scheme to increase the granularity of backoff. The scheme works on the principle of reacting less on the losses occurred in the beginning when packet is transmitted and react more as more losses are observed. Each transmission is termed as a state where each transmission corresponds to state 1, 2, 3, or 4. Each state number corresponds to number of retransmissions. When a new transaction is carried out, the transaction state is considered to be 1. This state is increased each time by one as retransmission is carried out. Similarly, whenever a packet is transmitted successfully without any retransmissions, the state is decreased by one. This way the backoff values are set accordingly. Optimization of backoff values can be performed by state values within the transaction as well as across multiple transaction.

A variable backoff factor is calculated for each value of state. For the backoff values and the formulas used to calculate these values; reader is referred to [23]. Unlike CoCoA’s two estimators (weak and strong), the proposed scheme has four levels of estimators. As the losses are increased and more transmissions are failed, higher percentage of backoff is added to the RTO calculation. The paper employs the following equation (Equation (2)) for the calculation of overall RTO:

(2) $$\begin{array}{c} RTO_{overall}=w*RTO_{obtained}+\left(1-w\right)*RTO_{overall} \end{array}$$

The parameter *w* here is the weight to the obtained RTO.

#### 4.1.5. Adaptive Congestion Control

The issue of ambiguity in the weak estimator values and for setting the appropriate values of constants for VBF and aging mechanism of RTO in order to decide whether and how to take the proper actions, is covered by the adaptive congestion control algorithm proposed in [4]. An option of transmission count is added to the CoAP message header to resolve the issue of weak estimator. In the case of retransmission of CON message, a new copy of the same message with different message ID is encapsulated. This helps in identifying the number of retransmission and therefore, the ambiguity whether an ACK message is from a transmission or a retransmission for weak estimator is resolved.

For making the VBF values adaptive to actual conditions of network, the algorithm considers RTOstrong as reference. Furthermore, the lower and upper thresholds are replaced by (1/3)*RTOstrong and (5/3)*RTOstrong respectively. The lower bound keeps RTO value to increase from RTOstrong until second retransmission while upper bound enables fast RTO to increase relatively. In case of default RTO value of 2 s, these values almost get back to the default values of 0.7 s and 3.3 s for lower and upper thresholds respectively.

The range for RTO value is set to (1/3*RTOstrong, 5/3*RTOstrong) in case of RTO aging and is adjusted in a forcing manner for when the value is out of range or not updated for longer periods of time.

#### 4.1.6. CoCoA+

Betzler et al. propose an advance congestion control mechanism (CoCoA+) in [7] as another solution for the issues in CoCoA. The CoCoA+ was proposed to overcome the shortcomings of CoCoA. As the weak RTT estimator in CoCoA is ambiguous and effects the overall RTO calculation, CoCoA+ proposes to reduce the impact of weak RTT estimator in the calculation of overall RTO by reducing the value of ‘K’ (the RTT variance multiplier) from 4 to 1.

(3) $$\begin{array}{c} RTO_{X}=RTT_{X}+K_{X}*RTTVAR_{X} \end{array}$$

Moreover, the weight of weak RTO estimator is limited in the overall RTO calculation. It is reduced from 50% to 25%.

(4) $$\begin{array}{c} RTO_{overall}=0.25*RTO_{weak}+0.75*RTO_{overall} \end{array}$$

In addition, for the weak RTT measurements, the CoCoA+ limits the measurements to be taken from first transmission and first retransmission only.

These solutions help in avoiding large increments in the overall RTO values. The CoCoA+ is yet unable to select the correct RTO value in case of burst traffic. This is caused due to inaccurate measurements of retransmitted RTT during bursty traffic, hence resulting in spurious retransmissions. In fact, Ancillotti and Bruno in [39] evaluated the congestion control performance of default CoAP and CoCoA+ and found out that in various network conditions, CoCoA+ performs significantly worse than default CoAP congestion control mechanism, such as in case of small RTT values and for bursty traffic.

#### 4.1.7. Improved Adaptive Congestion Control

The previously proposed methods do not consider the problem of choosing a right RTO value in case of burst traffic. In addition, these methods also do not consider the packet loss ratio. Packet loss ratio is defined as the number of packets received at the receiver end over number of packets sent by the sender. The packet loss ratio is used to evaluate the loss performance of method. [24] proposes an improved congestion control algorithm based on the packet loss ratio and RTT values of previous transmission. The method proposes two scenarios using packet loss ratio as key parameter and adjusts the RTO value accordingly based on the previous RTT values.

Case 1: The RTO value is updated according the formula presented in Equation (5) in case when packet loss ratio is lower than 50% in order to prevent unnecessarily long idle delays, whereas the RTO value is updated according to the formula presented in Equation (1) to correct the loss value.

(5) $$\begin{array}{c} RTO_{recent}=RTT*packetlossratio+\left(1-packetlossratio\right)*RTO_{previous} \end{array}$$

(6) $$\begin{array}{c} RTO_{recent}=RTO_{previous}*packet_{l}oss_{r}atio+\left(1-packet_{l}oss_{r}atio\right)*RTT \end{array}$$

Since the RTO value is updated in each transmission based on the packet loss ratio, there is no need for RTO aging mechanism. Calculation of RTO value in each transmission causes too much overhead and can cause delay in transmission.

#### 4.1.8. CACC

Context-Aware Congestion Control (CACC) proposed in [25] tackles the problem of differentiating the scenario of packet loss due bit error rate and congestion. It identifies the correct RTT of retransmitted message ACK by considering the dynamic network conditions. It comprises of three RTT estimators; weak RTT estimator, strong RTT estimator and failed RTT estimator. The strong and failed RTT combined together represent the successful deliver and packet dropping where the strong RTT is calculates in successful delivery and failed RTT is in the case of packet dropping. This scenario highlights the chance of link level packet collision, since some packets are delivered while rest are dropped. On the other hand, the high weak RTT value represents node level congestion delay. The method also restricts the RTO shrinkage in order to avoid negative variation in RTT which causes spurious retransmissions. In addition to this, the CACC also considers the retransmission count (RC) information in message transmission/retransmissions which enables it to accurately detect the weak RTT and smoothed RTT values. Finally, it incorporates the RTO aging mechanism for both small and large RTO estimations to avoid false RTO values in several network conditions. This aging technique is based on the CoCoA+ mechanism and it waits for the CACC to eventually increase the performance after RTO value is set to default in aging mechanism. This causes additional delays while waiting for CACC to increase performance over time. Moreover, the RTTVAR variable tends to vanish in case when sequence of similar RTTs is samples. This causes RTO values to get close to measure RTT. Also, in case of bursty traffic, neither the small weight of weak RTT (K = 1), not the avoidance of weak estimator is beneficial. Furthermore, the RTO value may increment steeply due to lack of aging mechanism of weak RTO.

#### 4.1.9. FASOR

This congestion control mechanism works in the case of bufferbloat condition and copes up with high link error rates. In Fast-Slow RTO (FASOR) [26], RTO computation is separated into two categories. Fast RTO computation is employed for unambiguous RTT samples while the Slow RTO computation is performed for ambiguous RTT samples to overcome deep bufferbloat and heavy congestion. This avoids extra delays and also helps under link errors by reducing flow completion.

For retransmission timer backoff, FASOR introduces a novel and self-adaptive timer containing three transitive states i.e., FAST / FAST-SLOW-FAST / SLOW-FAST. Each of these states have different backoff logic and it adapts to the dynamic network states. This enables FASOR to prevent extra power consumption due to unnecessary retransmissions and balance between the tradeoff of aggressive and conservative retransmissions.

The major issue with the proposed scheme is that it does not include special logic for senders remaining idle that is typical for CoAP. Moreover, the upper bound of slow RTO is kept 60 s, which can be improved further.

#### 4.1.10. pCoCoA

Bolettieri et al. highlight the issues of CoCoA+ in [8] and propose pCoCoA—a precise congestion control algorithm to solve these issues. The proposed mechanism is based on two major elements:

1. A method for linking requests to responses precisely even in case of retransmissions
2. Several modifications to the estimation algorithm of RTO

For linking the requests to responses precisely, the CoAP option of transmission counter (TC) is used. This links the ACK message of each transmission to its corresponding CON message. The TC value is updated even for retransmissions; hence it also detects spurious retransmissions. For the case of spurious retransmission, pCoCoA sets up a flag to consider it for future RTO computations.

To avoid vanishing of RTTVAR variable due to similar RTT sampling in a short period of time, max mean deviation of RTO is estimated. Therefore, issues because of sudden RTT variation are avoided by using max mean deviation which takes instead more time to decrease, thus decrease of final RTO value is limited. In addition, in the case of spurious transmissions, SRTO estimator grows faster due to increased weight of RTTVAR, which helps in limiting the successive spurious transmissions.

#### 4.1.11. CoCoA++

CoCoA++: Delay gradient-based congestion control: Another congestion control mechanism based on the gradient of RTT measurements over time is proposed by Rathod et al. in [27]. The proposed method provides remedies for the issues in congestion control in default CoAP, CoCoA, and its variants, such as CoCoA+. These methods use per packet RTT measurements to predict congestion in network, but these measurements are noisy and unreliable. CoCoA++, on the other hand, relies on CAIA Delay Gradient (CDG) [40] for the purpose of predicting network congestion by obtaining a gradient of RTT over time and provides a Probabilistic Backoff Factor (PBF) for controlling congestion in network.

With the use of delay gradient, the CoCoA++ eliminates the purpose of weak and strong RTO estimates. Also, unlike CoCoA and CoCoA+, the RTO value is not updated based on per packet RTT samples, instead the RTO is updated after receiving periodic information about the delay gradients from CDG. This enables CoCoA++ to not rely on VBF and hence it is replaced by PBF. The formula for calculating PBF is given in Equation (7).

(7) $$\begin{array}{c} PBF=\left\{\begin{array}{c} 1.42,P[backoff]>Xandg>0\\ 0.7,otherwise \end{array}\right. \end{array}$$

where *P[backoff]* is the probability of backoff that CDG returns, ‘X’ is the uniformly distributed random value and ‘g’ is the delay gradient. The probability of backoff is compared with the uniformly distributed random value ‘X’. The backoff is applied to RTO only in the case when we have positive rate of change of RTT. This is represented by the condition g > 0.

In the case of congestion, the PBF increases the RTO by a factor of 1.42 whereas it decreases the RTO by a factor of 0.7 for no congestion.

The issue with CoCoA++ is that with higher average packet sending rate, the subsequent retransmissions might occur quickly, causing the node to run out of retransmissions quickly.

#### 4.1.12. Genetic CoCoA++

Another congestion control algorithm based on CoCoA++ proposed by Yadav et al. in [28], in addition to problem of large changes in RTO estimates in CoCoA+, also mentions that congestion control algorithms that save previous states are not affordable for constrained IoT devices due to memory limitations. Therefore, they propose that employs CDG and Genetic Algorithm (GA) for RTO calculation for a fixed interval of time. The method uses RTT(min) instead of RTT(max) for calculation to gain better results. To identify the congestion in the network, difference between current and previous RTT(min) is used. RTT(min) is tracked for 5 s and this observation period, minimum value of RTT is selected from all the observed ones. After the selection, crossover is performed which is done considering the previous and new value of minimum RTT $\left(RTT_{m}in\right)$ and then the PBF is calculated according to CDG obtained from difference of previous and new RTT(min). Finally, it calculates RTO and continues the same process. Although it solves some issues of congestion control of CoAP and CoCoA+, but the 5 s observation time might not be enough for the case of underwater and mobile communication. Moreover, the proposed scheme does not consider burst traffic.

#### 4.1.13. Message Loss Feedback based

Congestion Control Scheme using Message Loss Feedback: A rate-based congestion control scheme was proposed in [29] to tackle the problem of congestion detection in default CoAP. For default CoAP, at least 2 out of 16 messages must be transmitted as CON messages for detection of congestion. To overcome this problem and detect the congestion in case of unreliable transmission as well, the proposed method defines a 1-bit field in the message header as ‘CS field’. The field value is either 0 or 1 depending on the type of transmitted message as either CON or NON respectively. For every transmission, the packet records the CS field in a ‘CS_list_S’ which is a list that holds the values of CS field for sender. On reception of a message, the receiver node updates the same list on receiver side called ‘CS_list_R’. The receiver list is then sent back to the sender with ACK in case of CON message. However, for the case of NON message loss, the number of packet loss is mentioned in the receiver side list i.e., CS_list_R and sends it to the sender in the next ACK message. The sender node compares the value in its list upon reception of ACK and subtracts it to find the packet loss number. This packet loss number is used to find the current transmission rate which in turn calculates the congestion value. In this way the proposed method detects congestion in network.

For congestion control, the method varies the transmission rate instead of congestion window size. It uses a token bucket mechanism for congestion control with the assumption that bucket size is not limited. When there is a data packet to be sent, it checks the remaining token size and transmits only if the token size is bigger than data size, otherwise it waits until a token with bigger size is created. The proposed method varies the packet transmission rate according to network congestion by adjusting the token generation rate. Although the approach performs better in terms of transmission success rate and throughput; however, it does not consider the scenario when number of CON and NON messages will be equal. In such case the loss rate of transmission cannot be found for CON messages.

#### 4.1.14. Content Freshness Based

A congestion control scheme is proposed in [30] which counters the congestion issue in network by controlling the size of congestion window in real time. The congestion window is varied based on the measured congestion ratio and freshness of congestion ratio information. Whenever a new ACK message is received by the sender node, the congestion ratio is measured. When the Congestion Ratio (CR) measured in previous ACK is greater than the current CR value, the congestion window size is reduced and when previous CR value is less than the current CR value the congestion window is increased to allow additional packet transmission. Moreover, the source node also measures the freshness of congestion ratio information by measuring the interval in addition to current CR value. This interval is the difference between reception time of current and previous ACK. If the interval is large the CR information is considered old. Finally, at the reception of the ACK message the source node also measures the RTT and distinguishes it as weak RTT (WRTT) or strong RTT (SRTT). If RTT is SRTT, the source node considers the update in current congestion window size as final congestion window size until the reception of next ACK message, whereas, if RTT is WRTT, the source divides it by 2 and determines if WRTT is greater than 2xSRTT. In case when WRTT is less than 2xSRTT, it is assumed that the network suffers low congestion and small reduction in congestion window is sufficient. However, in case when WRTT is greater than 2xSTT, high congestion in network is observed and congestion window needs to be reduced sufficiently.

#### 4.1.15. BDP-CoAP

BDP-CoAP proposes a rate-based congestion control method for CoAP [31] which is derived from the BBR (Bottleneck Bandwidth and Round-trip propagation time) protocol. BBR’s estimator of bottleneck bandwidth (BW) is redesigned to cope with the issues of lossy links and the short-term unfairness of channel in IoT networks. In case of short-term unfairness, a particular node in IoT network may acquire channel for short period of time in the time window and obtain high instantaneous delivery rates. This causes the BBR to overestimate the available bandwidth. BDP-CoAP uses an estimator that combines both max and min delivery rate measurements to derive bottleneck bandwidth estimates to avoid the overestimation of BW in case of short-term unfairness of channel access.

BDP-CoAP also tracks the number of missed bandwidth samples over the observation period, and it leverages this information to make the bottleneck BW estimator either more or less aggressive to vary the pace of transmission accordingly.

#### 4.1.16. CoAP-R

Another rate-based congestion control mechanism is proposed by Ancillotti et al. in [32] to tackle the performance issues of CoCoA in case of light and bursty traffic conditions and the problem of unfair bandwidth allocation in different traffic scenarios.

The proposed method is named as CoAP-R which uses the tree-based routing structure of IoT deployments to help in the discovery of bottleneck links. Using the information of bottleneck links, the proposed method employs a max-min fair allocation of available bandwidth in the network in a distributed manner. This process then regulates the message transmission rates of CoAP devices accordingly. However, since the proposed scheme is for tree-based routing structure, if a node is inactive in tree, it would miss the time when bandwidth allocation is performed. Also, with inactive node, link capacity estimation would not be possible and this would lead to wrong allocation of bandwidth among other nodes.

### 4.2. Enhanced CoAP for Interoperability

The authors in [41] propose a modified version of CoAP to address the issue of unavailability of cross protocol authentication between HTTP and CoAP. The proposed method, WoT-Auth, uses the HTTP Digest Access Authentication (HDAA) method to overcome the issue. HDAA contains a challenge and a response format. The ‘WWW-Authenticate’ HTTP header is used in transmission of HDAA challenge while ‘Authorization’ HTTP header is used in encoding of HDAA response. The content format of header includes the list of key-value pairs prefixed with Digest. The user distinguishes between the payload and authentication data using the CoAP ‘Options’. Given that the textual format for entries in WWW-Authenticate and Authorization HTTP headers is inefficient in terms of space and parsing them is also more complex, binary format is another choice to opt. However, for each entry a fixed size of field is not sufficient since some entries are of variable length for example realm and username. The CoAP ‘options’ not only allows variable length entries but also efficient encoding of these values. However, due to the availability of limited option numbers, modification is required in CoAP message format i.e., in CoAP ‘options’ value. This modification in CoAP is achieved by changing the CoAP ‘options’ parameter of CoAP message format. The paper proposes embedding of options inside the CoAP options value i.e., the inclusion of sub-options in a single ‘options’ parameter of message. This enables the implementation of encoding multiple HDAA challenge and response sets in single ‘options’ value of CoAP message. The proposed flexible encoding scheme is realized by implementing the multiple sub-options in CoAP options parameter in order to fit multiple length variables in single CoAP ‘options’ value. Each sub-option employs the same encoding scheme as in CoAP ‘options’.

### 4.3. Enhancement in CoAP Security

This subsection describes some enhancements made in the DTLS which is used for security in CoAP. Some of the enhancements made in DTLS by much research in different ways are mentioned below.

#### 4.3.1. Enhanced DTLS Protocol

The traditional DTLS initiates the process of authentication and authorization between a client and a server which is not secure as it is vulnerable to hacking attacks. An attacker acting as a client may send numerous ClientHello messages and hack the server in traditional DTLS, giving him access to private and sensitive data at server. The scheme proposed in [42], based on CoAP, presents an enhanced version of DTLS for end-to-end security for IoT in healthcare. The enhanced DTLS employs use of smart gateways for authentication and authorization, unlike traditional DTLS. After the process of authentication and authorization is complete, the gateway connects the client to the server using a session update. In the proposed approach, after the connection is established, the communication is performed between client and server via a smart gateway. With the help Advanced Encryption Standard-Counter with Cipher Block Chaining-Message Authentication Code (AESCCM) encryption and decryption algorithm, the data is securely transmitted between client and server. In the case when the user (client) may move out of the range of one smart gateway (say smart gateway-1), in order to maintain continuous communication, it uses neighbor solicitation and neighbor advertisement to identify another smart gateway (smart gateway-2) and shares all the necessary security information with it. Elliptic Curve Digital Signature Algorithm (ECDSA) is used to perform mutual authentication between smart gateway-1 and smart gateway-2. Afterwards, to authorize the end user to communicate with smart gateway-2, DTLS session update is carried out and continuous communication between client and server is realized securely.

#### 4.3.2. Enhanced Security with Hashing

The CoAP protocol is enhanced in terms of security in [43] where authors have focused on the integrity of CoAP messages. Instead of using DTLS security protocol, hash functions are employed as DTLS is not very efficient and requires high cost and computational power compared to the proposed hashing method. Hash function encryption provides one-way encryption. Similarly, the message that is hashed to some hash value cannot be decrypted back [44]. The hash functions are usually employed in the authentication of message for enhancing the security for example Message Authentication Code (MAC) [45]. The proposed method tests three hash functions including SHA-1, SHA224, and SHA256. The method is implemented on a home automation model consisting of sensors and controller. Keeping the same message format, the CoAP payload in CoAP messages is attached with sensor sequence-id value. The hash value is then calculated and attached with the payload and the message is sent to controller. On the controller side, the hash value of message is calculated where controller is synchronized with sensor’s clock. If the hash values match, further action is taken otherwise, message is discarded. The drawback of the proposed enhancement is that performing complex computation on sensors will eventually drain the sensors’ energy. Therefore, the energy consumption should also be considered.

Another method to secure privacy and data protection in lightweight IoT devices is proposed in [46]. Several symmetric key block ciphers for lightweight devices such as Advanced Encryption Standard (AES), Rivest Cipher 6 (RC6), Twofish, SPECK, ChaCha20-Poly1305, and Lightweight Encryption Algorithm (LEA) are evaluated in GCM (Galois/counter mode) mode with all supported key sizes. The evaluation is performed by measuring the metrics such as execution times, battery drainage and throughput. The test results exhibit the best performance of hardware-accelerated AES compared to all other algorithms with very good encryption throughput of 426.964 MiB/s with a 128-bit key; however, it is not suitable for devices with memory constraints. ChaCha20-Poly1305 is therefore a good adoption for lightweight devices with block ciphers such as SPECK and LEA according to the test results in [46].

### 4.4. Enhanced CoAP for Streaming Services

Owing to its lightweight capability, CoAP can be used for streaming services in constrained environments where devices have limited processing and power capabilities. For content delivery in CoAP, packets are sent using stop-and-wait method where a message carries some content from one node to another and then waits for the acknowledgement from receiver to confirm the content reception before sending more content in next message. The stop-and-wait method is not an efficient choice for streaming services as it involves large amount of data to be transmitted which exceeds the payload size of CoAP message. The upper bound of payload size in CoAP message is 1024 bytes only for limiting the communication overhead at IP layer due to packet fragmentation. Therefore, in order to use CoAP for streaming services, [47] proposes an extended version of CoAP. To overcome the inefficiency of stop-and-wait method of CoAP in streaming, the proposed scheme uses single request to retrieve multiple data blocks. This is beneficial for request messages in reducing the number of channel access. Therefore, the throughput performance can be increased. The number of data blocks that can be retrieved in a single request vary according to the network and channel conditions. In the case of stable network and no congestion, more packets can be sent, and higher throughput is achieved. The maximum number of data blocks that can be requested at a time is set to 8 in order to help in case of loss recovery. The paper also proposes a loss recovery method in case when data blocks are not received at the receiver due to lossy network environment.

## 5. Qualitative and Quantitative Analysis of Congestion Control Schemes

This section presents the qualitative and quantitative analysis of enhanced versions of congestion control mechanisms discussed in this paper. The analysis highlights the improvements in the enhanced schemes quantitatively and explains the use of scheme in different scenarios.

### 5.1. Qualitative Analysis

In this subsection, we discuss the enhanced congestion control schemes qualitatively. Various congestion control schemes are evaluated in different network topologies and different network traffic scenarios. Some schemes including CoCo-RED, CoCoA, 4-state estimator, adaptive congestion control scheme, and genetic CoCoA++ consider the continuous traffic scenarios, whereas some other schemes including improve adaptive, CACC, message loss feedback-based, and content freshness-based consider the constant traffic pattern. Finally, there are only a few schemes that consider the burst traffic pattern which include CoCo-RED, CoCoA, and CoCoA+. Compared to the default CoAP congestion control, the enhanced versions achieve better quality of network (QoN) in terms of various performance parameters. Based on our analysis, we can infer that the enhanced congestion control schemes such as message feedback-based, CoCoA, CACC, content freshness based, 4-state estimator, and adaptive congestion control scheme have higher throughput than the default CoAP congestion control with message feedback-based being the lowest among them and adaptive congestion control scheme being the highest one. In terms of settling time, CoCo-RED, CoCoA, and CoCoA+ analyze this metric and achive lower settling time than default CoAP. Therefore, CoCoA+ is suitable for the application where one requires lowest settling time. Another parameter i.e., packet delivery ratio can be used to identify the suitable scheme for the scenario where one needs to have higher packet delivery ratio. For example, if CoAP is to be applied in the IoUT environment, the CoCoA+ is better candidate then default CoAP as it achieves higher packet delivery ratio than default CoAP. Depending upon the requirement of application, different schemes can be implemented to improve the QoN. However, there is always a tradeoff between different performance parameters. For instance, if CACC is applied in the underwater sensor network, it will be suitable in terms of saving energy as it consumes less energy as compared to other schemes; however, the throughput will not be as high as that of adaptive congestion control scheme.

### 5.2. Quantitative Analysis

This subsection describes the quantitative analysis of congestion control schemes discussed in the paper. Most of the congestion control schemes discussed in the paper compare their performance to the default CoAP and the congestion control/advance i.e., CoCoA, while some compare it with CoCoA+. Table 3 shows the quantitative analysis of discussed congestion control schemes of CoAP. The schemes are evaluated with the following performance metrics: settling time, response time, packet loss ratio, throughput, end-to-end delay, network adaptability, energy consumption, and retransmission ratio. Among all the schemes, CoCo-RED, CoCoA, CoCoA+ consider the scenario of burst traffic while rest of them either consider constant, random or periodic network traffic. Moreover, the schemes are tested in different network topologies such as grid, dumbbell, chain, cross, random, and concentric circles. The last column of table presents the average improvement in different performance metrics of proposed schemes compared to default CoAP in percentages. The performance metrics of CoCoA++ and genetic CoCoA++ schemes are compared with the CoCoA only.

The quantitative analysis performed and presented in Table 3 can help us identify when it is better to use one solution or the other. For instance, if one wants to achieve higher throughput then they can adopt the adaptive congestion control mechanism as it achieves the highest average percentage of throughput (i.e., 56%) than other schemes compared to default CoAP. Similarly, if the target is to achieve low energy consumption instead, one can use CACC scheme as it achieves an average percentage improvement of 41% in energy consumption compared to default CoAP. However, in CACC, there is a tradeoff between energy consumption and throughput. Therefore, depending on the application requirement, one can choose between various schemes.

## 6. Open Challenges

Unlike HTTP CoAP operated over UDP and thus significantly reduces the overhead. However, the number of devices attached to the Internet is continuously increasing, and thus using the existing CoAP architecture may perform inefficiently in dense network scenarios. The CoAP is designed with keeping the message overhead as low as possible along with less usage of fragmentation option. However, still there exist a number of challenges and issues in the existing CoAP architecture. In this regard, we come up with the following main challenges existed in the current architecture of the CoAP.

### 6.1. Energy Efficiency of CoAP-Enabled Nodes

As we know, a CoAP node regularly checks the server for resource discovery. To discover a resource, CoAP implements two different approaches i.e., (1) Distributed Resource Discovery (DRD) and (2) Centralized Resource Directory (CRD) [48]. In the case of DRD, a CoAP-enabled device directly communicate with the server for the required resources. However, the CoAP-enabled node required high energy if a resource is not available for a longer time and the node operates on the battery power. Similarly, in the case of CRD to avail resources, CoAP-enabled nodes periodically communicating with a centralized directory. However, in the case of CRD, the directory should be updated regularly, otherwise, a CoAP-enabled node will consume its battery eventually. Such constant failure in getting resources increases the failure probability of the network. Resulting, in the degradation of the services provided by the network.

### 6.2. Interoperability

Enabling interoperability among different technologies is a challenging job. Similarly, with the design of the new protocol, it is important to build a mechanism for devices to speak to each other without technology barriers. The CoAP protocol is mostly based on the REST API and if, therefore, a device does not support CoAP may result in failure of communication. Therefore, the CoAP protocols support internetworking communication with HTTP. The REST-based style of CoAP enables it to communicate to the HTTP protocol over proxies that are specifically designed for enabling interoperability among CoAP and non-CoAP devices [49,50]. In general, it is easy to implement the mapping between CoAP and HTTP via proxies. However, it is still challenging whenever a CoAP-enabled device communicating with a non-CoAP and non-HTTP device. Various other technologies do not support HTTP and REST resulting in communication loss among the CoAP and those technologies.

### 6.3. Congestion Control

As CoAP is initially designed to cope with the congestion problem in IoT networks. However, as millions of devices join the IoT networks every year, it is therefore not possible to control the congestion in dense network scenarios. In this regard, other similar protocols exhibiting the properties of CoAP with some new feature is introduced [6]. Those protocols consist of CoCoA and CoCoA+. The new protocols somehow reduce the effect of congestion on the network. However, still, some challenges exist such as backward compatibility, real-time implementation, complex calculation of RTTs, etc. As the CoAP is designed for tiny devices such as sensors, motes, etc. therefore performing complex calculations periodically may drain the energy eventually. Therefore, it is necessary to design protocols considering the battery-powered nature of IoT devices.

### 6.4. Security of CoAP

The biggest challenge is to provide secure communication with high performance in constrained environments [51]. DTLS is considered to provide security in CoAP as it is based on the UDP. DTLS itself has limitations such as large message and handshake compression [52] and is not suitable for CoAP proxy modes [53]. The use of DTLS in constrained environments can be hindered as mentioned in [54], for instance, for message transmission, the defined timers in a protocol which may need large-sized buffers for holding data for retransmission on the receiver side, and the required size of code in order to support DTLS. Some solutions for identifying the cyber-attacks have been proposed by making the TLS/DTLS faster and flexible [55,56], nevertheless, there are still some issues that need to be tackled. In addition to providing secure communication among resource constrained devices, another challenging task is to secure the data privacy and protection. Numerous cryptography solutions are available to tackle this problem. In this regard, [46] discusses and evaluates several symmetric key algorithms for lightweight devices and provides their comparison with the proposed work. Moreover, the authors measured the several metrics such as execution times, battery drainage and throughput for all the tested ciphers and highlighted the old ciphers which are susceptible to more attacks compared to modern and optimized ciphers.

Furthermore, in the context of open research challenges in CoAP security, [57] mentions that the support of public keys and certificates can be explored further. Existent methods of Online Certification Status Protocol (OCSP) [58] or the OCSP stapling—TLS Certification Status Request extension mentioned in [59] can be applied by simplifying and modifying these mechanisms accordingly. For supporting multicast communication securely, the deficit of appropriate key management mechanisms is another important aspect to investigate. The authors in [60] mention two ways to design Group key management; One way to design group key management is integration with DTLS handshake in order to support key negotiation and another way is designing it externally to CoAP. Concerning the header compression mechanisms of DTLS [61], either existing implementations may be used to provide some support or new mechanisms need to be designed to map between DTLS and compressed DTLS. Security gateways can be used to support such mechanisms for providing secure end-to-end communications by mapping between TLS and DTLS. Besides, security gateways may also benefit from working against intrusion detection and attack tolerance mechanisms. For developing adequate methods for CoAP-based IoT communication, existing implementations on intrusion detection for sensor networks may be valuable [62,63,64].

## 7. Conclusions

The CoAP developed for the lightweight communication in IoT domains for the constrained devices has been applied in many fields ranging from home automation to disaster and health management systems. The use of CoAP in more applications has exhibited the shortcomings in CoAP architecture such as inefficient congestion control mechanism, poor interoperability with other devices, etc. This led to many enhancements in the basic CoAP according to specific applications; for example, many advance congestion control mechanisms were developed over time to improve the issues in the default congestion control mechanism. Similarly, enhancements in other operations such as security, interoperability, etc. were also made. This survey paper presents some applications where CoAP is implemented in various domains and covers in detail the enhancements in congestion control mechanism as well as some application specific enhancements over time. A tabular comparison is provided for the congestion control mechanisms discussed in the survey. In addition, the survey also provides a qualitative and quantitative analysis of the enhanced congestion control mechanisms of CoAP. the qualitative and quantitative analysis can help the researchers to identify when it is better to use one scheme or another, depending on the application requirements. After discussing the enhancements in the basic architecture of CoAP, the survey discusses the open research challenges in CoAP implementation in the fields of security, interoperability, resource discovery, energy efficiency and congestion control. The current architecture of CoAP may perform inefficiently in the future as the number of internet devices are continuously increasing. Therefore, the highlighted research gaps provide insight to future research directions in order to enable CoAP for performing better in the dense network scenarios.

## Figures and Tables

**Table 1:** Applications of CoAP

Paper	Role of CoAP	Application
Z. Mi et al. [9]	Direct compatibility of medical sensors with internet + interaction of sensors with other nodes using RESTful communication	Interoperability
B. Oryema et al. [10]	Implementation of messaging system with MQTT model using CoAP	Interoperability
S.-y. Ge at al. [11]	Design and implementation of healthcare platform with IEEEE 11073 PHD	Interoperability
W. Li et al. [12]	Integration of two healthcare standards ISO/IEEE 11073 and IHE PCD-01 for communicating between medical IoT devices	Integration
Viel et al. [13]	Integration of CoAP with Open Smart Grid Protocol (OSGP) for information exchange between devices in smart grids (SG)	Integration
D. Garcia-Carrillo et al. [14]	Integration between AAA infrastructures and EAP	Authentication and Authorization
M. B. Tamboli et al. [15]	To provide communication with packets having low overhead in CoAP-based authentication and access control framework for IoT	Authentication and Access Control
P. Krawiec et al. [16]	For delivering the media segments to consumers in implementing dynamic streaming over CoAP	Streaming Services
W. ur Rahman et al. [17]	To perform adaptive streaming for constrained wireless environments	Video Streaming
T. L. Scott et al. [18]	Transfer of data from IoT nodes to cloud	Cloud Computing Services
S. R. Jan et al. [19]	Observing resources (temperature values) in IoT environment and WSNs	Resource Observation
B. Djama et al. [20]	For advertising and demanding of resource directories using CoAP REST methods	Resource Discovery
D. Ugrenovic et al. [21]	Implementation of a remote healthcare monitoring system using CoAP client/server model	Real-time Remote Monitoring

**Table 2:** Comparison of Congestion Control Mechanisms.

Scheme Name	Adopted Mechanism	Comm. Type	Burst Traffic Support	Complexity Level	Issues Countered	Shortcomings
Default CoAP [2]	Binary Exponential Backoff (BEB)	Reliable	No	Low	Basic Congestion Control	Idle delays between retransmissions, does not consider dynamic network
CoCo-RED [22]	Buffer management using Revised Random Early Detection	Reliable	Yes	Low	Reduced packet loss and response time of network	Fixed backoff values, does not consider dynamic network conditions
CoCoA [6]	RTT measurements + Variable Backoff	Reliable	No	Low	Basic congestion control issues + RTO aging	Ambiguity in weak RTT estimator
4-state Estimator [23]	State0based Variable Backoff	Reliable	No	Medium	Idle delays between successive (re)transmissions in CoCoA	Not included
Adaptive Congestion Control [4]	Transmission count-based	Reliable	No	Low	Ambiguity in weak estimator values	More bandwidth, and energy is consumed in solving ambiguity of weak estimator especially for wireless communication
CoCoA+ [7]	Modifications in CoCoA RTO calculations	Reliable	No	High	Ambiguity in weak estimator values of CoCoA	Inaccurate measurement of retransmitted RTT in burst traffic
Improved Adaptive Congestion Control [24]	Packet loss ratio-based RTO calculations	Reliable	Yes	Low	Congestion Control in burst traffic, elimination of RTO aging mechanism	Additional overhead in calculating RTO in each transmission, poor adaptability in RTO
CACC [25]	RTT Estimators + Retransmission Count based	Reliable	No	High	Differentiating the scenario of packet loss due bit error rate and congestion	Poor RTO aging mechanism, vanishing of RTTVAR variable for similar consecutive RTT samples, lack of aging mechanism for weak RTO causing steep RTO increment
FASOR [26]	RTO Estimators + State-based backoff logic	Reliable	No	Medium	Bufferbloat condition, high link error rates	No special logic for senders remaining idle
pCoCoA [8]	Transmission Count + Modifications in RTO Estimation + Estimation of Max mean deviation of RTO	Reliable	Yes	High	Spurious retransmissions, vanishing RTTVAR due to similar RTT sampling	Not included
CoCoA++ [27]	Delay Gradient based calculation + probabilistic backoff	Reliable	No	High	Shortcomings of default CoAP, CoCoA, CoCoA+	Sender runs out of retransmissions due to quick retransmissions caused by increased packet sending rate
Genetic CoCoA++ [28]	CoCoA++-based + Genetic algorithm	Reliable	No	High	Issues of CoCoA+ including: (i) Accurate retransmission RTO measurement (ii) Large changes in RTO estimates	RTT observation time is limited, burst traffic is not considered
Message Loss Feedback-based [29]	Message Loss Feedback-based	Reliable/Unreliable	No	Low	Congestion detection in default CoAP	Loss rate of transmission cannot be found if number of CON and NON messages are equal
Content Freshness based [30]	Congestion window size control	Reliable/Unreliable	No	Medium	Congestion Control in default CoAP	Loss rate of transmission cannot be found if number of CON and NON messages are equals
BDP-CoAP [31]	Estimation of bottleneck bandwidth	Reliable	Yes	Medium	Issues of lossy links + short term unfairness of channel	Not included
CoAP-R [32]	Rate-based	Reliable	Yes	Medium	Performance issues of CoCoA in light and burst traffic + unfair bandwidth allocation	Scenario of inactive/malfunctioned node is not considered

**Table 3:** Quantitative Analysis of Enhanced Congestion Control Schemes.

Scheme Name	Performance Metrics	Topology	Traffic Scenarios	Avg Percentage Improvement
CoCo-RED [22]	Settling time, response time, packet loss	Chain, grid, cross, dumbbell, random	Continuous, burst	Settling time: 2%, Response time: 8%, Packet loss: 21%
CoCoA [6]	Throughput, settling time	Not mentioned	Continuous, burst	Throughput: 19Settling time: 26.67%
4-state Estimator [23]	Throughput, goodput	Not mentioned	Continuous	Throughput: 19% Goodput: 40%
Adaptive Congestion Control [4]	Throughput	Not mentioned	Continuous	Throughput: 56%
CoCoA+ [7]	packet delivery ratio, end-to-end delay, settling time	Chain, dumbbell, grid	Periodic, burst, mixed	Packet delivery ratio: 4.4% End-to-end delay: 18.5% Settling time: 27.5%
Improved Adaptive Congestion Control [24]	number of dropped packets, number of successful transactions	Point to multipoint	Constant	Dropped packets: 40.8% Successful transactions: 7.5%
CACC [25]	Throughput, end-to-end delay, energy consumption	Grid, chain, dumbbell	Constant	Throughput: 21.8% End-to-end delay: 47.8% Energy consumption: 41%
CoCoA++ [27]	Number of packets transmitted, average packet sending rate	Grid, flower, dumbbell, chain	Periodic	Number of packets transmitted: 15% Avg packet sending rate: 5.2%
Genetic CoCoA++ [28]	Packet failure rate	Grid, dumbbell	Continuous	Packet failure rate: 32.14%
Message Loss Feedback-based [29]	Average packet reception ratio, throughput	Random	Constant	Packet reception ratio: 3.215% Throughput: 18.54%
Content Freshness based [30]	Throughput, number of message transmissions	Random	Constant	Throughput: 23.46% Number of transmissions: 31.95%

## Abbreviations

The following abbreviations are used in this manuscript:

IETF	Internet of Engineering Task
CoAP	Constrained Application Protocol
IoT	Internet of Things
IoUT	Internet of Underwater Things
IoE	Internet of Everything
WSN	Wireless Sensor Networks
REST	Representational State Transfer
UDP	User Datagram Protocol
TCP	Transmission Control Protocol
BEB	Binary Exponential Backoff
CoCoA	Congestion Control/Advance
RTT	Round Trip Time
CON	Confirmable
NON	Non-confirmable
RTO	Retransmission Timeout
DTLS	Datagram Transport Layer Security
MQTT	Message Queuing Telemetry Transport
OSGP	Open Smart Grid Protocol
SG	Smart Grids
COIIoT	CoAP and OSGP Integration for the Internet of Things
PED	Pending Event Descriptor
AAA	Authentication, Authorization and Accounting
EAP	Extensible Authentication Protocol
LP-WAN	Low-Power Wide Area Networks
LO-CoAP-EAP	Low-Overhead CoAP-EAP
DASCo	Dynamic Streaming over CoAP
DASH	Dynamic Streaming over HTTP
JSON	JavaScript Object Notation
RD	Resource Directory
PRD	Proactive RD Discovery
FDR	Fully distributed push-pull Resource Discovery
CoCo-RED	Congestion Control Random Early Detection
RevRED	Revised Random Early Detection
FPB	Fibonacci Pre-Increment Backoff
BMT	Buffer Management Technique
VBF	Variable Backoff Factor
CACC	Context-Aware Congestion Control
RC	Retransmission Count
FASOR	Fast-Slow RTO
TC	Transmission Counter
CDG	CAIA Delay Gradient
PBF	Probabilistic Backoff Factor
GA	Genetic Algorithm
CR	Congestion Ratio
WRTT	Weak RTT
SRTT	Strong RTT
BBR	Bottleneck Bandwidth and Round-trip propagation time
BW	Bandwidth
HDAA	HTTP Digest Access Authentication
AESCCM	Advanced Encryption Standard-Counter with Cipher Block Chaining-Message Authentication Code
ECDSA	Elliptic Curve Digital Signature Algorithm
MAC	Message Authentication Code
AES	Advanced Encryption Standard
RC6	Rivest Cipher 6
LEA	Lightweight Encryption Algorithm
GCM	Galois/counter mode
DRD	Distributed Resource Discovery
CRD	Centralized Resource Directory
OCSP	Online Certification Status Protocol
PFR	Packet Failure Rate
QoN	Quality of Network
